# An isochronous stress ratio logarithmic strain curve based clay creep model considering the effects of hardening and damage

**DOI:** 10.1038/s41598-024-56488-2

**Published:** 2024-03-25

**Authors:** Peng Wang, Yin Tang, Peng Ren, Hua Zhang

**Affiliations:** 1 Geotechnical Engineering Institute, Sichuan Institute of Building Research, Chengdu, 610081 China; 2 Sichuan Engineering Research Center for Mechanical Properties and Engineering Technology of Unsaturated Soils, Chengdu University, Chengdu, 610106 China; 3 School of Architecture and Civil Engineering, Chengdu University, Chengdu, 610106 China

**Keywords:** Clay, Creep test, Isochronous stress ratio-logarithmic strain curve, Creep mechanical feature point, Creep model, Solid Earth sciences, Civil engineering

## Abstract

Creep is one of the typical mechanical properties of clay, and studying the creep mechanical properties of clay is of great significance to construction projects in clay sites. This study conducted creep tests on Chengdu clay and found that the soil mass underwent elastic deformation, decay creep deformation, steady-state creep deformation, and accelerated creep deformation. The isochronous stress ratio-logarithmic strain curves and their mathematical models were proposed to thoroughly analyze clay creep mechanical properties. Creep automatic feature points, such as linear elastic extreme point, initial yield point, long-term strength point, and plastic point, were identified on the curve. Considering the hardening and damage effects during creep loading, linear elastic and viscoelastic elements considering the time-dependent damage, a viscoplastic element considering the load hardening effect, and viscoplastic and plastic elements considering the load damage effect were established based on the element model and the Riemann–Liouville fractional derivative. Based on the mechanical properties of the whole clay creep process, the creep mechanical feature points, and the established element model, a clay creep model was proposed considering the hardening and damage effects. The rationality and regularity of the creep model were verified using the creep test data. This research accurately revealed the creep mechanical properties of clay and facilitated soil deformation prediction, thus providing technical guidance and references for construction projects in clay sites.

## Introduction

As one of the essential mechanical properties of geotechnical materials, rheology reflects the stress and strain relationship of geotechnical materials over time, mainly including creep, stress relaxation, and long-term strength. The clay creep properties reflect the strain–time relationship under constant loading stress and significantly impact the construction projects in clay sites^1–4^. Therefore, studying clay creep is of great practical significance.

Due to the wide distribution of clay, it often appears in bridges, tunnels, airports, ports, highways, buildings, and other projects. In the construction and long-term operation processes of these projects, the creep of clay will significantly affect their safety. Therefore, the creep test of clay can deeply understand the timeliness of clay and provide effective technical parameters for the design, construction, operation, and maintenance of related projects. The conventional creep test methods for clay include the direct shear creep test^5,6^ and the triaxial creep test^7–9^. Ding et al.^10^ studied the creep mechanical properties of soft clay under different over-consolidation ratios through triaxial creep tests and found that the over-consolidated soft clay only included decay creep and steady-state creep stages but not the accelerated creep stage. Long et al.^11^ studied the creep mechanical properties of red clay under different confining pressures, loading stresses, and drainage conditions through triaxial creep tests. They found that the creep deformation increased with decreased confining pressure, increased loading stress, or drainage. Microscopic experiments showed that the soil exhibited decreased pore volume and pore area during the creep process and reached a new structural balance. Zhu et al.^12^ studied the effects of soil density and structure on the creep coefficient by one-dimensional direct shear creep tests and found that the creep coefficient had a simple nonlinear relationship with porosity. Through triaxial creep tests on Shanghai silt clay, Tang et al.^13^ found the steady-state loading stress threshold and failure loading stress threshold in the creep process, which divided the creep process into a gradually stable deformation stage, a rapidly increasing deformation stage, and a failure stage. Yu et al.^14^ conducted creep tests lasting about one year and found that clay had a high creep potential, its creep rate did not always increase with the increase of stress, and the creep properties of clay were closely related to the loading stress path.

Plotting the isochronous stress–strain curve data is shared for creep mechanical property analysis^15–17^. Mechanical parameters such as initial elastic modulus and long-term strength under creep conditions can be determined based on isochronous stress–strain curves^18,19^. However, due to defects such as the heavy reliance on creep loading level, the mechanical parameters derived from isochronous stress–strain curves are not accurate enough to directly contribute to relevant research and practical projects^20^.

Clay rheological models generally include empirical models^21–23^ and element models. The elastic, viscous, and plastic elements are the three essential elements of the element model^24^, which can be combined to form viscoelastic and viscoplastic models^25^. Fractional calculus has been applied to describe clay creep mechanical properties as it accurately describes the stress history of viscoelastic materials^26,27^. Fractional calculus is often combined with viscous elements to form fractional calculus viscous elements, which are combined with other elements to form fractional calculus creep models^28–32^.

Although the current research on clay creep mechanical properties and models has yielded fruitful results, the following deficiencies remain^33–35^: (1) A method to accurately determine the various clay creep stages is yet to be found in the existing research on clay creep mechanical properties; (2) Under the influence of time and stress during creep loading, clay exhibits internal hardening and damage effects simultaneously, but few studies simultaneously considered the creep hardening and damage effects of clay, warranting in-depth investigations.

Based on the existing research, this study conducted creep tests on Chengdu clay to determine its creep mechanical properties. To thoroughly analyze the stress–strain patterns under creep conditions, isochronous stress ratio-logarithmic strain curves and their mathematical models were proposed. Based on the curve properties and clay creep mechanical properties, five creep automatic feature points were identified on the curve, which accurately distinguished the various clay creep mechanical stages and laid the foundation for building element models and creep models. Considering that the soil structure may tend to stabilize or fail after the initial yield, A viscoplastic element considering the load hardening effect and a viscoplastic element considering the load softening effect were established, and linear elastic, viscoelastic, and plastic elements considering the time-dependent damage were also constructed. Based on the clay creep mechanical properties and the established elements, a clay creep model considering the hardening and damage effects was proposed. The model could accurately describe the mechanical properties of the whole creep process of clay and provide technical guidance and references for construction projects in clay sites.

## Clay creep test

### Specimen properties

**Figure 1:**
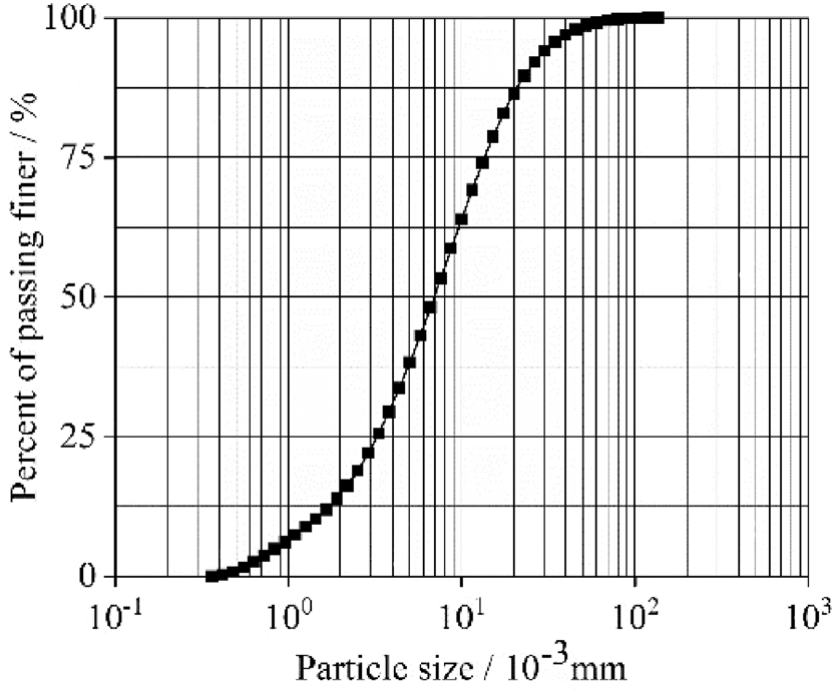
Grading curve of unsaturated expansive soil.

The creep test specimens were collected from a clay site in Chengdu at a sampling depth of 5 m. The soil specimens' main physical and mechanical properties are shown in Table 1, and their grading curves are presented in Fig. 1.

**Table 1:** Physical and mechanical properties of Chengdu clay.

Specific gravity	Water content/%	Density/g m^−3^	Liquid limit/%	Plastic limit/%
2.76	23.0	2.04	43.90	19.90

### Test methods

**Figure 2:**
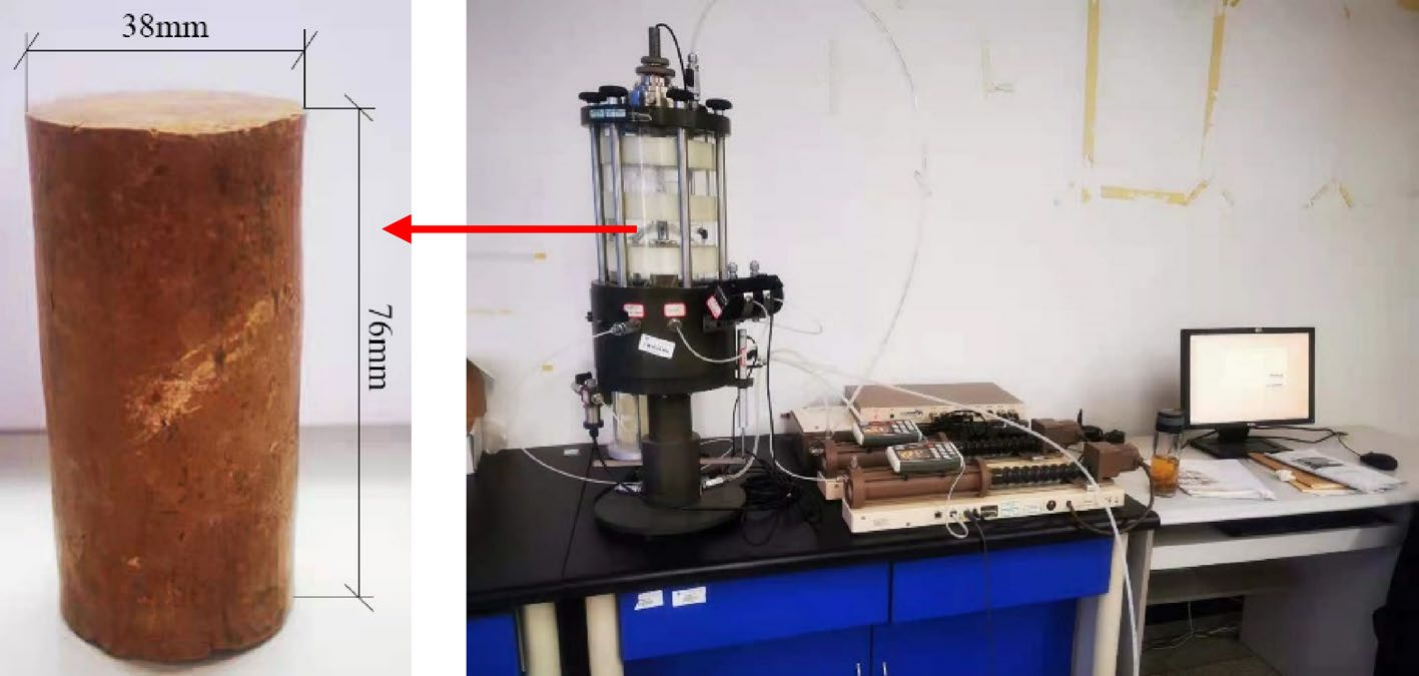
Chengdu clay creep test.

The creep tests were conducted on a GDS triaxial test system. The specimens were prepared as follows: (1) The specimens were 38 mm in diameter and 76 mm in height, and apply petroleum jelly to the gasket in contact with the sample; (2) The specimens were consolidated under 100 kPa confining pressure, which was loaded at a rate of 1 kPa/min, and the consolidation condition was that the pore water pressure dissipation of over 95%; (3) Creep tests were carried out using the stepwise loading method, and the deviator stress path was* q* = 20 kPa, 38 kPa, 55 kPa, 75 kPa, 90 kPa, 110 kPa, 128 kPa, and 145 kPa, with the deviator stress loading rate of 1 kPa/min. Figure 2 shows the Chengdu clay creep test.

### Experimental result analysis

**Figure 3:**
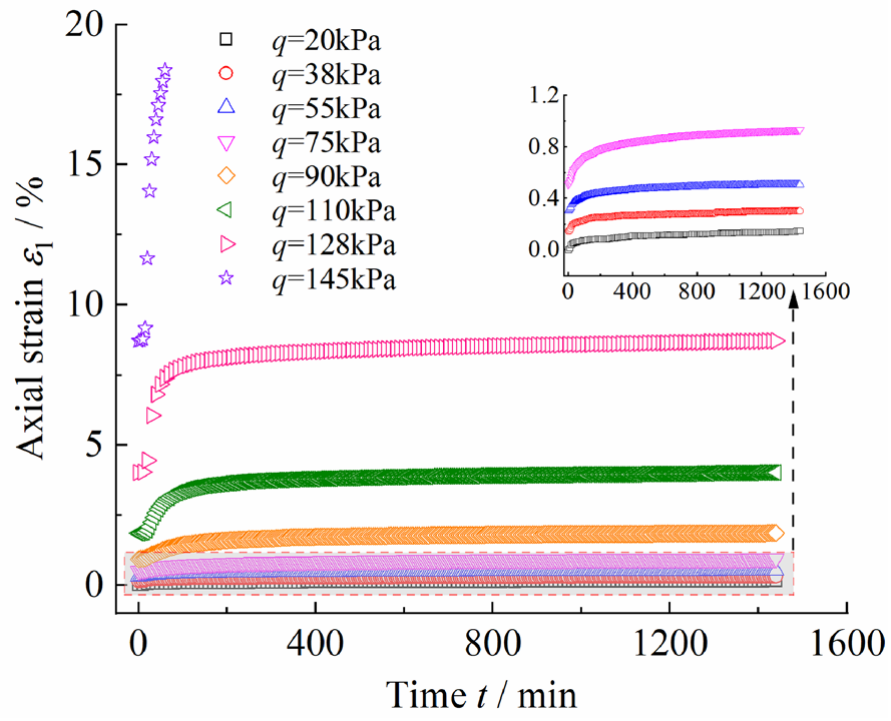
Chengdu clay creep test curve.

The creep test curve of Chengdu clay is shown in Fig. 3. It can be observed that when* q* ≤ 75 kPa, the clay creep strain is less than 1.2%. At this time, the strain includes the transient strain and the creep strain that gradually stabilizes, and the clay is in the decay creep stage. When *q* ≥ 145 kPa, the clay creep deformation increment accelerates until the failure of the specimen, at which time the clay is in the accelerated creep stage. When 75 kPa < *q* < 145 kPa, the clay gradually transitions from decay creep to accelerated creep, covering the steady-state creep stage. Therefore, the strain of Chengdu clay under creep test conditions mainly included transient strain and creep strain, and the creep process included decay creep, steady-state creep, and accelerated creep stages.

## Stress–strain relationship under isochronous conditions

### Isochronous stress–strain curve

**Figure 4:**
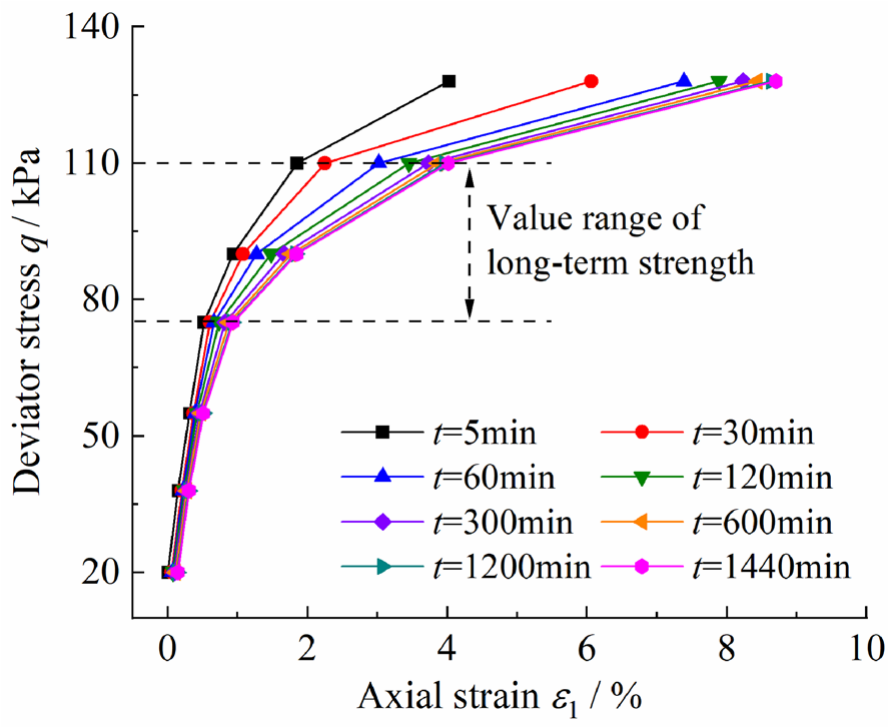
Isochronous stress–strain curve.

According to the creep test curves, the isochronous stress–strain curves are plotted in Fig. 4. It can be observed that when* q* ≤ 75 kPa, the isochronous stress–strain curves are roughly linear; When *q* > 75 kPa, the isochronous stress–strain curves gradually deflect to the strain axis, showing significant nonlinear characteristics; Meanwhile, the nonlinearity of the curves is more significant over time.

The value at the inflection point of the isochronous stress–strain curve is generally taken as the long-term strength^19^. Significant inflection points can be observed on the isochronous stress–strain curves in Fig. 4 at *q* = 75 kPa and* q* = 110 kPa. Therefore, the long-term strength *q*_L_ determined based on the isochronous stress–strain curve is 75 kPa to 110 kPa, which is 51.72% to 75.86% of the maximum loading stress in the creep tests, with a range of about 24%. Thus, the wide range of long-term strength determined based on the isochronous stress–strain curve makes it difficult to determine the long-term strength accurately.

### Isochronous stress ratio-logarithmic strain curve

To accurately determine the creep mechanical characteristic values of clay, such as the long-term strength, the patterns of the isochronous stress–strain curve must be thoroughly analyzed. To this end, the stress and strain under isochronous conditions are transformed, as expressed in Eq. (1):

1 $$\left\{ {\begin{array}{*{20}l} {q_{r} = \frac{q}{{q_{f} }}} \\ {\varepsilon_{q} = - \ln (\varepsilon_{1} /100)} \\ \end{array} } \right.$$

where *ε*_1_ and* q* are the axial strain and deviator stress on the isochronous stress–strain curve, respectively; *q*_*f*_ is the creep failure load; *q*_*r*_ and *ε*_*q*_ are the stress ratio and logarithmic strain under isochronous conditions, respectively, with 0 < *q*_*r*_ ≤ 1.

**Figure 5:**
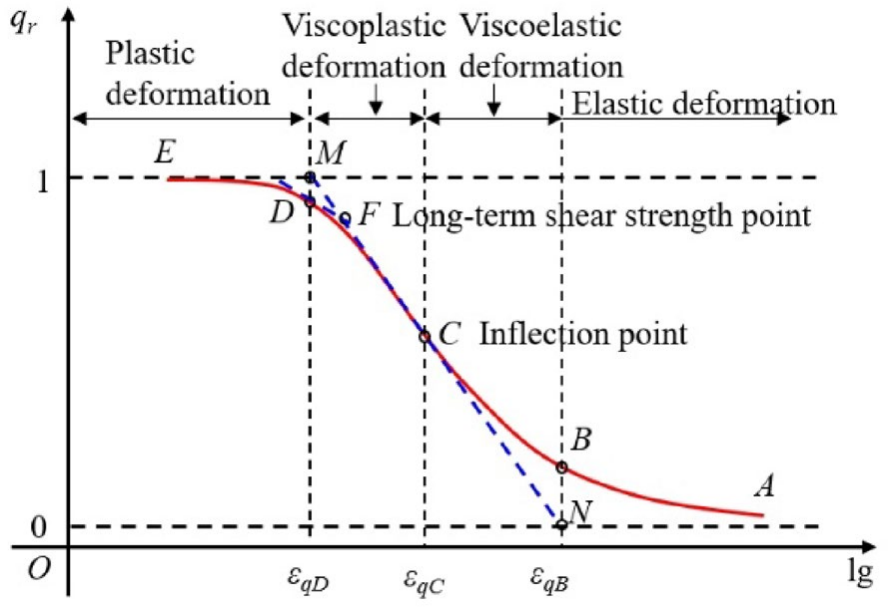
Isochronous stress ratio-logarithmic strain curve.

The isochronous stress–strain curve is converted into the isochronous stress ratio-logarithmic strain curve according to Eq. (1), and the converted curve is placed into the half-logarithmic coordinate system space, as shown in Fig. 5. It can be observed that the isochronous stress ratio-logarithmic strain curve has the following properties: (1) The curve has an inflection point *C*, and the *ABC* segment of the curve is concave, while the *CDE* segment is convex; (2) The upper boundary of the curve is *q*_*r*_ = 1, and the lower boundary is *q*_*r*_ = 0. With $\lim_{{q_{r} \to 1}} \varepsilon_{q} { = 0}$, $\varepsilon_{1} { = + }\infty$, indicating that the soil mass reaches a state of complete plastic deformation or failure at this time; When $\lim_{{q_{r} \to 0}} \varepsilon_{q} { = + }\infty$,$\varepsilon_{1} { = }0$, indicating that the soil mass is in a fully elastic state at this time.

According to the properties of the isochronous stress ratio-logarithmic strain curve, the tangent to the curve at point* C* intersects with the upper and lower boundaries at points* M* and* N*, respectively. The perpendiculars through points *N* and* M* intersect with the curve at points *D* and *B,* respectively, and the tangent at point* D* intersects segment *MN* through point *F.* According to the properties of the isochronous stress ratio-logarithmic strain curve and the mechanical properties of the whole creep process of the soil mass, points *B*,* C*,* D*, and *F* on the curve are defined as the creep mechanical feature points. Among them, point* B* is the linear elastic extreme point, point* C* is the initial yield point, point* D* is the complete yield point, and point* F* is the long-term strength point. Therefore, the *AB* segment on the isochronous stress ratio-logarithmic strain curve is a linear elastic stage describing the elastic deformation; The *BC* segment on the curve is the viscoelastic stage mainly representing the viscoelastic deformation in the decay creep stage; The *CD* segment on the curve is a viscoplastic stage primarily representing the viscoplastic deformation in the decay creep and steady-state creep stages; The *DE* segment on the curve is the plastic deformation stage mainly representing the deformation in the accelerated creep stage.

Isochronous stress ratio-logarithmic strain curve model.

According to phenomenology, the isochronous stress ratio-logarithmic strain curve is highly similar to the soil–water characteristic curve. Therefore, the VG model of the soil–water characteristic curve is used as the mathematical model of the isochronous stress ratio-logarithmic strain curve, as expressed in Eq. (2):

2 $$q_{r} = \left[ {1 + (k\varepsilon_{q} )^{n} } \right]^{ - m}$$

where *k*,* n*, and *m* are the model fitting parameters, *m* = 1–1/*n*.

To solve each characteristic point of the isochronous stress ratio-logarithmic strain curve with Eq. (2), the first- and second-order derivatives of Eq. (2) are derived, respectively, and the results are expressed in Eqs. (3) and (4).

3 $$K = q_{r}^{\prime } = \frac{{dq_{r} }}{{d\lg (\varepsilon_{q} )}} = - \frac{{\ln (10)nm(k\varepsilon_{q} )^{n} }}{{[1 + (k\varepsilon_{q} )^{n} ]^{m + 1} }}$$

4 $$q_{r}^{\prime \prime } = \frac{{dq_{r}^{\prime } }}{{d\lg (\varepsilon_{q} )}}{ = }\frac{{\ln^{2} (10)n^{2} m(k\varepsilon_{q} )^{n} \left[ {m(k\varepsilon_{q} )^{n} - 1} \right]}}{{[1 + (k\varepsilon_{q} )^{n} ]^{m + 2} }}$$

At the inflection point C, we have $q_{rC}^{\prime \prime } = 0$, and the coordinates of point* C* can be obtained from Eq. (4), as expressed in Eq. (5).

5 $$\left\{ {\begin{array}{*{20}l} {\varepsilon_{qC} = \frac{1}{{km^{1/n} }}} \\ {q_{rC} = \frac{1}{{\left( {1 + 1/m} \right)^{m} }}} \\ \end{array} } \right.$$

The slope* K*_C_ of the tangent at point* C* can be derived from Eqs. (5) and (3), as expressed in Eq. (6).

6 $$K_{C} = q_{r}^{\prime } \left| {_{{\varepsilon_{q} = \varepsilon_{qC} }} } \right. = - \frac{\ln (10)n}{{(1 + 1/m)^{m + 1} }}$$

The equation for the curve's tangent at point* C* can be derived from Eqs. (5) and (6), as expressed in Eq. (7).

7 $$q_{rC} (\varepsilon_{q} ) = (1 + 1/m)^{ - m} - \frac{\ln (10)n}{{(1 + 1/m)^{m + 1} }}\left[ {\lg (\varepsilon_{q} ) - \lg (1/(km^{1/n} ))} \right]$$

The abscissa of point* M* and the coordinates of point* D* can be obtained by substituting *q*_*r*_ = 1 into Eq. (7), as expressed in Eq. (8).

8 $$\left\{ {\begin{array}{*{20}l} {\varepsilon_{qD} { = }\varepsilon_{qM} = \frac{1}{k}\left( {\frac{{\xi_{D} }}{m}} \right)^{1/n} } \\ {q_{rD} = \left( {1 + \frac{{\xi_{D} }}{m}} \right)^{ - m} } \\ \end{array} } \right.$$

where $$\xi_{D} = 10^{{\frac{(1 + m)}{{\ln (10)m}}\left[ {1 - (1 + 1/m)^{m} } \right]}}$$.

The abscissa of point *N* and the ordinates of point* B* can be obtained by substituting *q*_*r*_ = 0 into Eq. (7), as expressed in Eq. (9).

9 $$\left\{ {\begin{array}{*{20}l} {\varepsilon_{qB} { = }\varepsilon_{qN} = \frac{1}{k}\left( {\frac{{\xi_{B} }}{m}} \right)^{1/n} } \\ {q_{rB} = \left( {1 + \frac{{\xi_{B} }}{m}} \right)^{ - m} } \\ \end{array} } \right.$$

where $$\xi_{B} = 10^{{\frac{1 + m}{{\ln (10)m}}}}$$.

The equation for the tangent at point* D* can be derived from Eqs. (3) and (8), as expressed in Eq. (10).

10 $$q_{rD} (\varepsilon_{q} ) = \left( {1 + \frac{\xi }{m}} \right)^{ - m} - \frac{\ln (10)n\xi }{{(1 + \xi /m)^{m + 1} }}{\kern 1pt} \left[ {\lg (\varepsilon_{q} ) - \lg \left[ {\frac{1}{k}\left( {\frac{\xi }{m}} \right)^{1/n} } \right]} \right]$$

According to Eqs. (7) and (10), the coordinates of point *F* can be derived, as expressed in Eq. (11).

11 $$\left\{ {\begin{array}{*{20}l} {\varepsilon_{qF} = 10^{{\frac{{\left( {q_{rD} - q_{rC} } \right) + \left[ {K_{C} \lg \left( {\varepsilon_{qC} } \right) - K_{D} \lg \left( {\varepsilon_{qC} } \right)} \right]}}{{K_{C} - K_{D} }}}} } \\ {q_{rF} = q_{C} + K_{C} \left[ {\frac{{\left( {q_{rD} - q_{rC} } \right) + \left[ {K_{C} \lg \left( {\varepsilon_{qC} } \right) - K_{D} \lg \left( {\varepsilon_{qD} } \right)} \right]}}{{K_{C} - K_{D} }} - \lg \left( {\varepsilon_{qC} } \right)} \right]} \\ \end{array} } \right.$$

The long-term strength *q*_L_ can be obtained from Eqs. (1) and (11).

### Chengdu clay creep test data analysis

**Figure 6:**
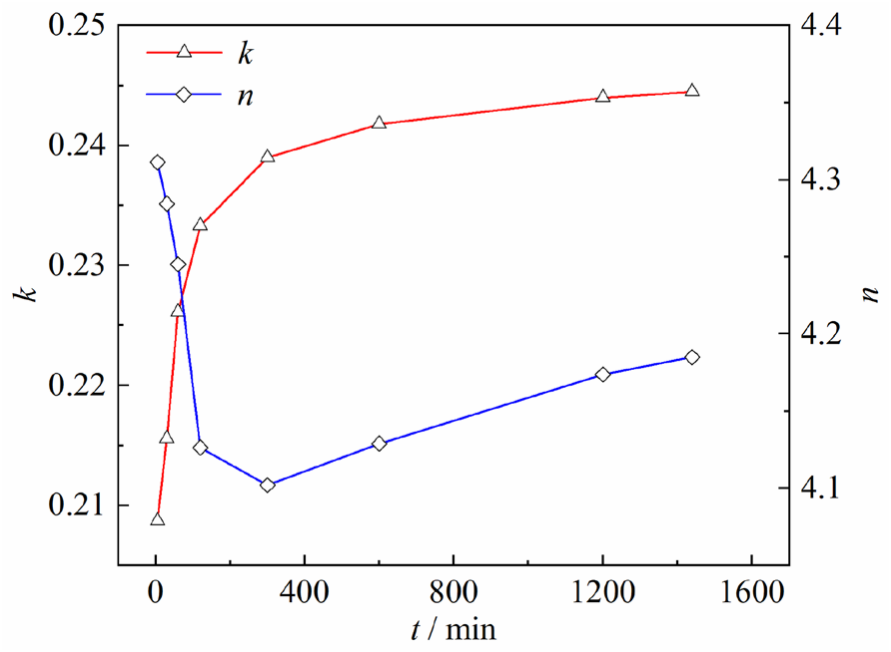
Calculation results of parameters* k* and* n.*

**Figure 7:**
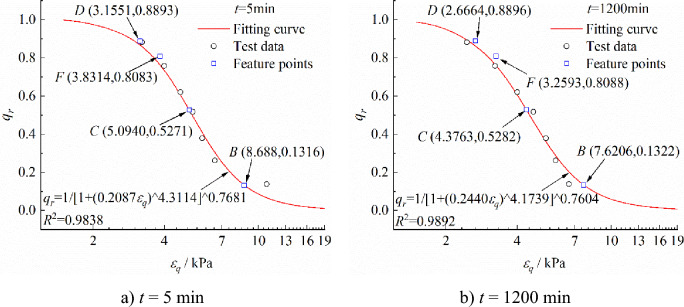
Results of Chengdu clay creep test data analysis based on isochronous stress ratio-logarithmic strain curve.

The isochronous stress–strain curves in Fig. 4 are converted and analyzed according to Eqs. (1) to (11). The fitting results of* k*,* n*, and *m* are presented in Fig. 6. Limited by the length of the article, the isochronous stress ratio-logarithmic strain curves and each creep mechanical feature points at* t* = 5 min and *t* = 1200 min are presented in Fig. 7. It can be observed from Fig. 6 that the parameter* k* increases linearly first with time and then increases nonlinearly; The parameter* n* decreases linearly at a larger rate first with time and then increases linearly at a lower rate. As shown in Fig. 7, the isochronous stress ratio-logarithmic strain test results have a fitting coefficient over 0.98 with their model, indicating a high degree of agreement between the test results and the model. Meanwhile, the calculation results of each mechanical feature point are clear.

**Figure 8:**
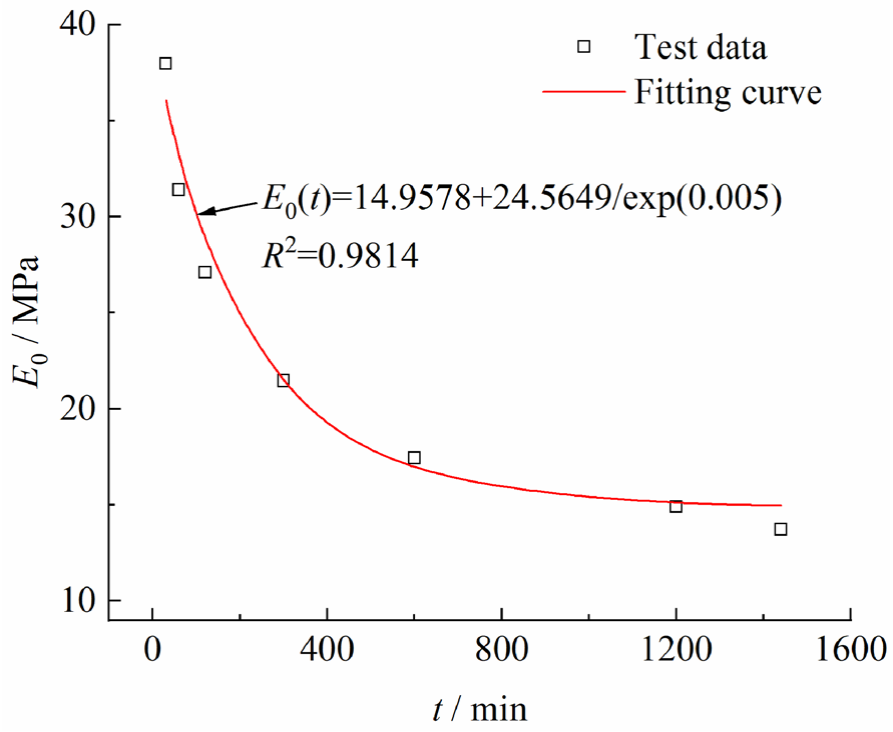
Elastic modulus fitting curve of the clay in the linear elastic stage.

According to Fig. 7, the elastic limit stress ratio *q*_*rB*_ is between 0.1316 and 0.1322, and the deviator stress is about 19 kPa. This study selects the creep data under the loading level of* q* = 20 kPa to calculate the elastic modulus of the clay in the linear elastic stage, and the results are shown in Fig. 8. It can be observed that with the increase of time, the linear elastic modulus drastically decreases first and then slowly decreases until stabilization. In this study, the relationship between the linear elastic modulus and time fitted using Eq. (12). The fitting results are shown in Fig. 8. According to the fitting results in Fig. 8, the long-term elastic modulus of Chengdu clay *E*_∞_ is 14.9578 MPa in the elastic deformation stage,* E*_0_ = 39.5227 MPa, and the parameter* w* is 0.005.

12 $$E_{0} \left( t \right) = E_{\infty } + \frac{{\left( {E_{0} - E_{\infty } } \right)}}{{\exp \left( {wt} \right)}}$$

where *E*_0_ and *E*_∞_ are the initial and long-term elastic modulus, respectively; *w* is the fitting parameter.

## Clay creep model

### Riemann–Liouville fractional derivative

Fractional calculus is an important method to study viscoelastic materials. This study proposes to establish creep model elements using the Riemann–Liouville fractional derivative. According to the Riemann–Liouville theory^18,19^, we have the following. Let the function $u$ be continuously integrable on (0, + ∞), and we have the following by finding its fractional integral over *t* ≥ 0 and Re(*n*) ≥ 0:

13 $$\frac{{{\text{d}}^{ - \alpha } [u(t)]}}{{{\text{d}}t^{ - \alpha } }}{ = }{}_{{t_{0} }}D_{t}^{ - \alpha } u(t) = \frac{1}{\Gamma (n)}\int_{{t_{0} }}^{t} {(t - \xi )}^{\alpha - 1} u(\xi )d\xi$$

where $\Gamma (n) = \int_{0}^{\infty } {t^{n - 1} } e^{ - t} dt$ is the Gamma function, and* α* is the fractional order.

The Riemann–Liouville fractional differential can be defined as follows: Let $u \in C$, $\gamma > 0$, and* m* be the smallest integer greater than *γ*, we have the following by letting *γ* = *β*-*α*:

14 $$\frac{{{\text{d}}^{\alpha } [u(t)]}}{{{\text{d}}t^{\alpha } }}{ = }{}_{{t_{0} }}D_{t}^{\alpha } u(t) = \frac{{{\text{d}}^{\beta } }}{{{\text{d}}t^{\beta } }}\left[ {{}_{{t_{0} }}D_{t}^{ - \gamma } u(t)} \right]$$

If *u*(*t*) is integrable in the vicinity of *t* = 0, and 0 ≤ *n* ≤ 1, the Laplace transform of the fractional calculus is:

15 $$\left. \begin{gathered} L\left[ {{}_{0}D_{t}^{ - \alpha } u(t),p} \right] = p^{ - \alpha } \overline{u} (p){\kern 1pt} {\kern 1pt} {\kern 1pt} {\kern 1pt} {\kern 1pt} {\kern 1pt} {\kern 1pt} {\kern 1pt} \hfill \\ L\left[ {{}_{0}D_{t}^{\alpha } u(t),p} \right] = p^{\alpha } \overline{u} (p){\kern 1pt} {\kern 1pt} {\kern 1pt} {\kern 1pt} {\kern 1pt} {\kern 1pt} {\kern 1pt} {\kern 1pt} \hfill \\ \end{gathered} \right\}$$

where *u*(*p*) is the Laplace transform of *u*(*t*).

### Elastic element considering time-dependent damage

The elastic element is used to describe the deformation pattern of the clay in the elastic stage. The linear elastic element is expressed in Eq. (16).

16 $$\sigma { = }E_{0} \left( t \right)\varepsilon_{{\text{e}}}$$

where *σ* is the loading deviator stress,* σ* = *q*, and *ε*_e_ is the linear elastic strain.

According to Eqs. (12) and (16), the linear elastic element model considering the time-dependent damage can be expressed in Eq. (17).

17 $$\varepsilon_{{\text{e}}} { = }\frac{\sigma }{{E_{\infty } + \left( {E_{0} - E_{\infty } } \right)\exp \left( { - wt} \right)}}$$

### Fractional viscoelastic element considering initial threshold

With *σ*_e_ < *σ* ≤ *σ*_y0_ (*σ*_e_ is the extreme value of linear elastic stress, and* σ*_y0_ is the initial yield strength), the clay has not yet yielded and is in the decay creep state. At this time, the viscoelastic element starts to work. The viscoelastic element consists of a linear elastic element and a fractional viscous element in parallel, and its model is expressed in Eq. (18).

18 $$\sigma_{{{\text{ve}}}} { = }\sigma - \sigma_{{\text{e}}} { = }E_{1} \varepsilon_{{{\text{ve}}}} {(}t{)} + \eta_{1} \frac{{{\text{d}}^{{\alpha_{1} }} \varepsilon_{{{\text{ve}}}} {(}t{)}}}{{{\text{d}}t^{{\alpha_{1} }} }}$$

where *σ*_ve_ is the stress of the viscoelastic element; *σ*_e_ is the extreme value of the linear elastic stress, which can be determined by the feature point *B* on the isochronous stress ratio-logarithmic curve; *ε*_ve_ is the strain of the viscoelastic element; *E*_1_ and* η*_1_ are the elastic modulus and viscosity coefficient of the viscoelastic element, respectively;* α*_1_ is the fractional operator of the viscoelastic element.

Through the Laplace transform, the viscoelastic element model can be obtained from Eq. (18), as expressed in Eq. (19).

19 $$\varepsilon_{{{\text{ve}}}} { = }\frac{{\sigma { - }\sigma_{{\text{e}}} }}{{E_{1} }}E_{{ - \alpha_{1} ,1}} \left( { - \frac{{\eta_{1} }}{{E_{1} }}t^{{ - \alpha_{1} }} } \right)$$

where $$E_{\mu ,\nu } (Z) = \sum\nolimits_{k = 0}^{\infty } {\frac{{z^{k} }}{\Gamma (\mu k + \nu )}}$$.

### Viscoplastic elements considering initial yield strength hardening effect

With *σ*_y0_ < *σ* ≤ *σ*_L_ (*σ*_L_ is the long-term strength), the soil mass enters the yield stage. Yet, the soil mass deformation eventually stabilizes, and the structure rebalances. Therefore, the soil structure exhibits load hardening, and the soil mass is in the decay creep stage at this time.

In the initial yield-load hardening stage of the soil mass, the viscoplastic element considering the load hardening effect consists of a fractional viscous element and a plastic element in parallel, and its model is expressed in Eq. (20).

20 $$\sigma_{{{\text{vpH}}}} = \sigma - \sigma_{{\text{y}}} \left( t \right){ = }\eta_{2} \frac{{{\text{d}}^{{\alpha_{2} }} \varepsilon_{{{\text{vpH}}}} {(}t{)}}}{{{\text{d}}t^{{\alpha_{2} }} }}$$

where *σ*_vpH_ and *ε*_veH_ are the stress and strain of the viscoplastic element considering the hardening effect,* η*_1_ is the viscosity coefficient of the viscoplastic element, and* α*_1_ is the fractional operator. *σ*_y_(*t*) is the yield strength of the soil mass after hardening over time.

Suppose *σ*_y_(*t*) obeys the hardening relationship in Eq. (21).

21 $$\sigma_{{\text{y}}} \left( t \right) = \sigma_{{{\text{y}}0}} + H\left( {\sigma - \sigma_{{{\text{y}}0}} } \right)t^{1 - \lambda }$$

where *σ*_y0_ is the initial yield strength, which can be determined by point* C* on the isochronous stress ratio-logarithmic curve; *H* and* λ* are the material parameters, and 0 ≤ *H* < 1, 0 < *λ* < 1.

The viscoplastic element model considering the load hardening effect can be derived from Eqs. (20) and (21), as expressed in Eq. (22).

22 $$\varepsilon_{{{\text{vpH}}}} { = }\frac{{\sigma - \sigma_{{{\text{y}}0}} }}{{\eta_{2} }}\left[ {\frac{{t^{{\alpha_{2} }} }}{{\Gamma \left( {1 + \alpha_{2} } \right)}} - \frac{{H\Gamma (2 - \lambda )t^{{1 + \alpha_{2} - \lambda }} }}{{\Gamma (2 + \alpha_{2} - \lambda )}}} \right]$$

### Viscoplastic element considering soil mass strength damage effect

With *σ*_L_ < *σ* ≤ *σ*_a_ (*σ*_a_ is the creep failure stress), the soil mass deformation after yielding increases at a steady rate, while the soil structure sustains damage until its failure. At this time, the soil mass is in a steady-state creep stage.

Kachanov's definition of the creep damage variable is expressed in Eq. (23).

23 $$\frac{{{\text{d}}D}}{{{\text{d}}t}} = A\left( {\frac{\sigma }{1 - D}} \right)^{{\beta_{1} }}$$

where *D* is the damage variable of the nonlinear element, and* A* and* β*_1_ are the material parameters.

The damage variable can be obtained by finding the integration of Eq. (23), as expressed in Eq. (24).

24 $$D\left( t \right){ = }1 - \left( {\frac{{t_{{\text{f}}} - t}}{{t_{{\text{f}}} - t_{{\text{L}}} }}} \right)^{{ - \beta_{1} - 1}}$$

where *t*_*f*_ and *t*_*L*_ are the start times of accelerated creep and steady-state creep, respectively, which can be determined by points* D* and* F* on the isochronous stress ratio-logarithmic strain curve, with $t_{f} - t_{L} = 1/[A(1 + n)\sigma^{ \wedge } \beta 1]$.

Based on the Lemaitre strain equivalence principle, the relationship between engineering stress and effective stress can be expressed in Eq. (25).

25 $$\sigma { = }\sigma^{\prime } \left( {1 - D} \right)$$

where *σ* and* σ*′ are the engineering stress and the effective stress, respectively.

In this study, the proposed steady-state creep-stage nonlinear damage element model is expressed in Eq. (26).

26 $$\sigma^{\prime} - \sigma = \eta_{3} \frac{{{\text{d}}\varepsilon_{{{\text{vp}}D}} (t)}}{{{\text{d}}t}}$$

where *ε*_*vpD*_ is the damaging strain and *η*_3_ is the viscosity coefficient at the steady-state creep stage.

The nonlinear damage element model for the steady-state creep stage can be obtained from Eqs. (24) to (26), as expressed in Eq. (27).

27 $$\varepsilon_{{{\text{vp}}D}} = \frac{\sigma }{{\eta_{3} }}\left[ {\frac{{\left( {\beta_{1} + 1} \right)\left( {t_{{\text{f}}} - t_{L} } \right)}}{{\beta_{1} }}\left[ {1 - \left( {\frac{{t_{{\text{f}}} - t}}{{t_{{\text{f}}} - t_{{\text{L}}} }}} \right)^{{ - \beta_{1} - 1}} } \right] - \left( {t - t_{{\text{L}}} } \right)} \right]$$

### Nonlinear accelerated creep element

With *σ*_a_ < *σ*, the soil mass exhibits accelerated deformation until its failure and is in an accelerated creep stage. The accelerated creep pattern is described with a fractional viscous element, the model of which is expressed in Eq. (28).

28 $$\varepsilon_{{\text{a}}} = \frac{{\sigma - \sigma_{{\text{a}}} }}{{\eta_{4} }}\frac{{t^{{\alpha_{3} }} }}{{\Gamma (1 + \alpha_{3} )}}$$

where *ε*_a_,* α*_3_, and* η*_3_ are the strain, fractional operator, and viscosity coefficient of the nonlinear accelerated creep element, respectively, with 1 < *α*_3_. *σ*_a_ is the creep failure deviator stress, which can be determined by point* D* on the isochronous stress ratio-logarithmic strain curve.

**Figure 9:**
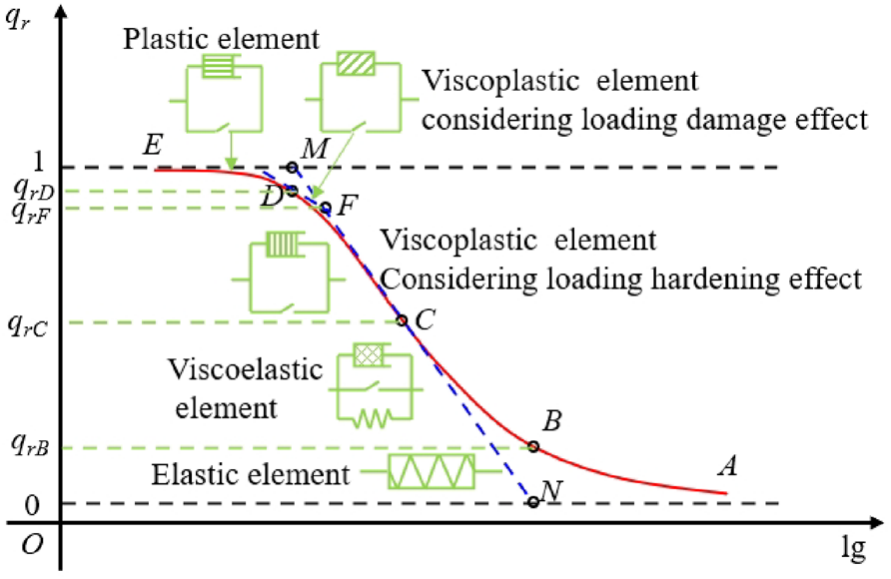
Element model of different creep mechanical property intervals on the isochronous stress ratio-logarithmic strain curve.

Finally, the creep mechanics interval of each element model established on the isochronous stress ratio-logarithmic strain curve is shown in Fig. 9.

## Clay creep model and validation

### Creep model

**Figure 10:**
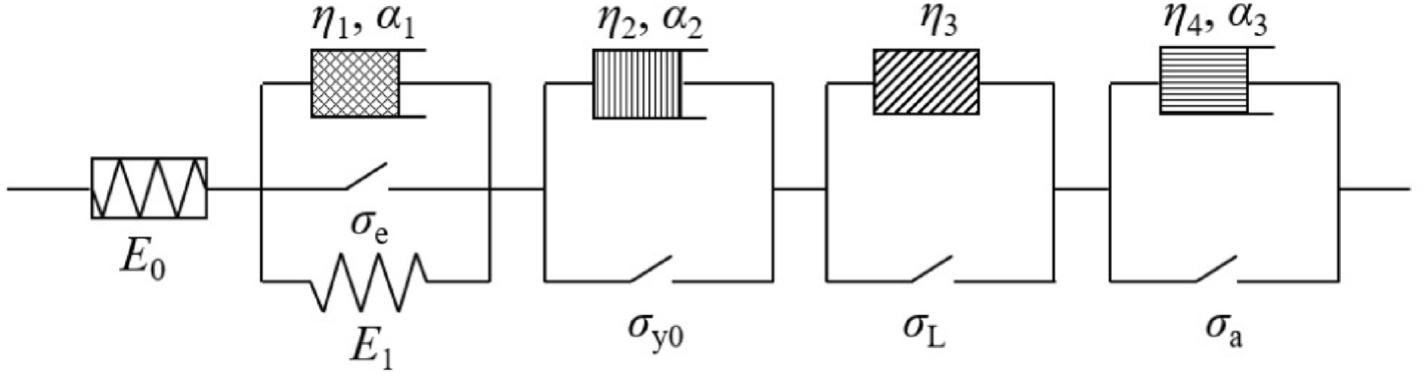
Clay creep model considering the effects of load hardening and damage.

According to the creep properties of clay and the established element model, the clay creep model proposed in this study is shown in Fig. 10. The clay creep model is as follows.

1. With 0 < *σ* ≤ *σ*_e_, only the elastic element undergoes deformation at this time, and the strain model is expressed in Eq. (29).
  29 $$\varepsilon \left( t \right){ = }\frac{\sigma }{{E_{0} \left( t \right)}}$$
2. With *σ*_e_ < *σ* ≤ *σ*_y0_, the viscoelastic element begins to deform, and the creep model is expressed in Eq. (30).
  30 $$\varepsilon \left( t \right){ = }\frac{\sigma }{{E_{0} \left( t \right)}} + \frac{{\sigma - \sigma_{{\text{e}}} }}{{E_{1} }}E_{{ - \alpha_{1} ,1}} \left( { - \frac{{\eta_{1} }}{{E_{1} }}t^{{ - \alpha_{1} }} } \right)$$
3. With *σ*_y0_ < *σ* ≤ *σ*_L_, the viscoplastic element considering the hardening effect begins to deform, and the creep model is expressed in Eq. (31).
  31 $$\varepsilon \left( t \right){ = }\frac{\sigma }{{E_{0} \left( t \right)}} + \frac{{\sigma - \sigma_{{\text{e}}} }}{{E_{1} }}E_{{ - \alpha_{1} ,1}} \left( { - \frac{{\eta_{1} }}{{E_{1} }}t^{{ - \alpha_{1} }} } \right) + \frac{{\sigma - \sigma_{{{\text{y}}0}} }}{{\eta_{2} }}\left[ {\frac{{t^{{\alpha_{2} }} }}{{\Gamma \left( {1 + \alpha_{2} } \right)}} - \frac{{H\Gamma (2 - \lambda )t^{{1 + \alpha_{2} - \lambda }} }}{{\Gamma (2 + \alpha_{2} - \lambda )}}} \right]$$
4. With *σ*_L_ < *σ* ≤ *σ*_a_, the viscoplastic element considering the damage effect begins to deform, and the creep model is expressed in Eq. (32).
  32 $$\begin{gathered} \varepsilon \left( t \right){ = }\frac{\sigma }{{E_{0} \left( t \right)}} + \frac{{\sigma - \sigma_{{\text{e}}} }}{{E_{1} }}E_{{ - \alpha_{1} ,1}} \left( { - \frac{{\eta_{1} }}{{E_{1} }}t^{{ - \alpha_{1} }} } \right) + \frac{{\sigma - \sigma_{{{\text{y}}0}} }}{{\eta_{2} }}\left[ {\frac{{t^{{\alpha_{2} }} }}{{\Gamma \left( {1 + \alpha_{2} } \right)}} - \frac{{H\Gamma (2 - \lambda )t^{{1 + \alpha_{2} - \lambda }} }}{{\Gamma (2 + \alpha_{2} - \lambda )}}} \right] \hfill \\ {\kern 1pt} {\kern 1pt} {\kern 1pt} {\kern 1pt} {\kern 1pt} {\kern 1pt} {\kern 1pt} {\kern 1pt} {\kern 1pt} {\kern 1pt} {\kern 1pt} {\kern 1pt} {\kern 1pt} \frac{\sigma }{{\eta_{3} }}\left[ {\frac{{\left( {\beta_{1} + 1} \right)\left( {t_{{\text{f}}} - t_{L} } \right)}}{{\beta_{1} }}\left[ {1 - \left( {\frac{{t_{{\text{f}}} - t}}{{t_{{\text{f}}} - t_{{\text{L}}} }}} \right)^{{ - \beta_{1} - 1}} } \right] - \left( {t - t_{{\text{L}}} } \right)} \right] \hfill \\ \end{gathered}$$
5. With *σ*_a_ < *σ*, the nonlinear accelerated creep element begins to deform, and the creep model is expressed in Eq. (33).
  33 $$\begin{gathered} \varepsilon \left( t \right){ = }\frac{\sigma }{{E_{0} \left( t \right)}} + \frac{{\sigma - \sigma_{{\text{e}}} }}{{E_{1} }}E_{{ - \alpha_{1} ,1}} \left( { - \frac{{\eta_{1} }}{{E_{1} }}t^{{ - \alpha_{1} }} } \right) + \frac{{\sigma - \sigma_{{{\text{y}}0}} }}{{\eta_{2} }}\left[ {\frac{{t^{{\alpha_{2} }} }}{{\Gamma \left( {1 + \alpha_{2} } \right)}} - \frac{{H\Gamma (2 - \lambda )t^{{1 + \alpha_{2} - \lambda }} }}{{\Gamma (2 + \alpha_{2} - \lambda )}}} \right] \hfill \\ {\kern 1pt} {\kern 1pt} {\kern 1pt} {\kern 1pt} {\kern 1pt} {\kern 1pt} {\kern 1pt} {\kern 1pt} {\kern 1pt} {\kern 1pt} {\kern 1pt} {\kern 1pt} {\kern 1pt} \frac{\sigma }{{\eta_{3} }}\left[ {\frac{{\left( {\beta_{1} + 1} \right)\left( {t_{{\text{f}}} - t_{L} } \right)}}{{\beta_{1} }}\left[ {1 - \left( {\frac{{t_{{\text{f}}} - t}}{{t_{{\text{f}}} - t_{{\text{L}}} }}} \right)^{{ - \beta_{1} - 1}} } \right] - \left( {t - t_{{\text{L}}} } \right)} \right] + \frac{{\sigma - \sigma_{{\text{a}}} }}{{\eta_{4} }}\frac{{t^{{\alpha_{3} }} }}{{\Gamma (1 + \alpha_{3} )}} \hfill \\ \end{gathered}$$

### Model validation

The proposed model in this study is verified using the Chengdu clay creep test data. Based on Figs. 6 and 7, the stress calculation results of the isochronous stress ratio-logarithmic strain curve feature points are expressed in Eq. (34), where *t*_L_ = 8640 min, and *t*_a_ = 11,520 min.

34 $$\left\{ {\begin{array}{*{20}l} {\sigma_{{\text{e}}} { = }\sigma_{B} { = }19.15{\kern 1pt} {\kern 1pt} {\text{kPa}}} \\ {\sigma_{{{\text{y0}}}} { = }\sigma_{C} = 76.57{\kern 1pt} {\kern 1pt} {\text{kPa}}} \\ {\sigma_{{\text{L}}} = \sigma_{F} = 117.26{\kern 1pt} {\kern 1pt} {\text{kPa}}} \\ {\sigma_{{\text{a}}} { = }\sigma_{D} = 129.001{\kern 1pt} {\kern 1pt} {\text{kPa}}} \\ \end{array} } \right.$$

**Figure 11:**
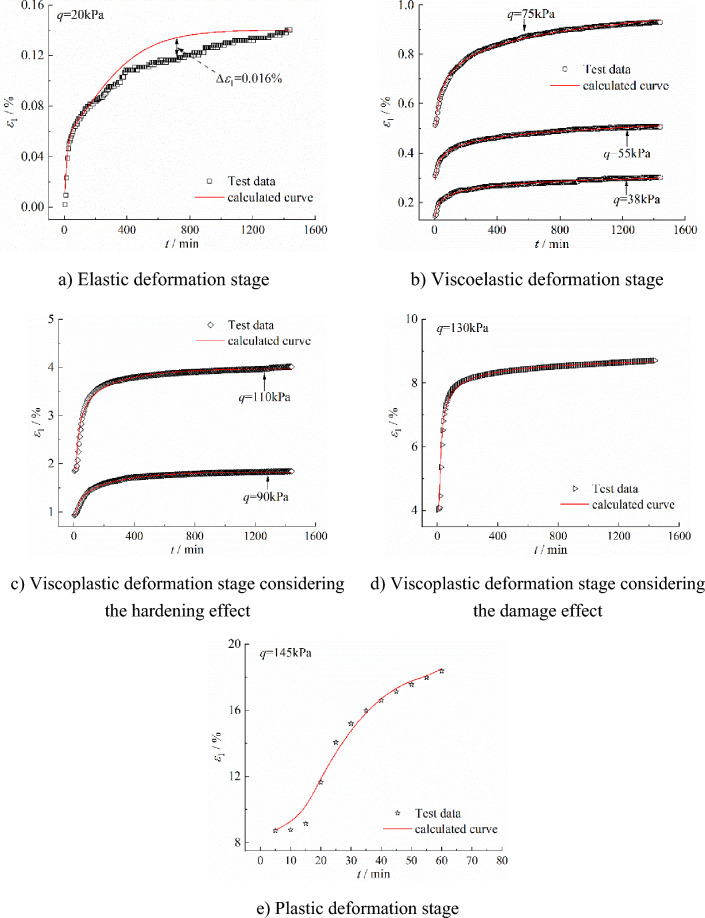
Comparison between the test results and the calculated results with the proposed creep model in the various creep stages of Chengdu clay.

The creep model proposed in this paper is used to calculate the creep stages of Chengdu clay. The calculation parameters are listed in Table 2, and the comparison between the model calculation results and the creep test results is shown in Fig. 11.

**Table 2:** Calculation parameters of Chengdu clay in the viscoelastic deformation stage.

*q*	*E*_1_	*η*_1_	*α*_1_	*η*_2_	*α*_2_	*H*	*λ*	*η*_3_	*β*_*1*_	*η*_4_	*α*_3_
38	1.914	2.026	0.009	–	–	–	–	–	–	–	–
55	1.301	1.327	0.005	–	–	–	–	–	–	–	–
76	1.087	1.106	0.006	–	–	–	–	–	–	–	–
90	1.449	1.453	0.001	2.178	0.413	0.981	0.997				
110	2.639	11.214	0.381	3.249	0.399	0.881	0.984				
130	1.305	1.717	0.071	0.363	0.021	0.655	0.973	3.3*10^9^	0.307		
145	1.995	1.515	0.100	1.111	0.917	0.999	0.951	8.2*10^8^	0.117	1.998	1.03

*q* is in kPa in the table; *E*_1_ is in MPa; *η*_1_,* η*_2_, and *η*_3_ are in MPa·min.

It can be observed that in the elastic deformation stage, the model calculation results are basically consistent with the test results. Between the loading time *t* interval of 700 min to 1300 min, the calculated strain is greater than the tested strain, and the maximum difference is 0.016%. In each deformation stage after the elastic deformation stage, the model calculation results are highly consistent with the creep test results, which verifies the rationality and applicability of the proposed creep model.

## Discussion

**Figure 12:**
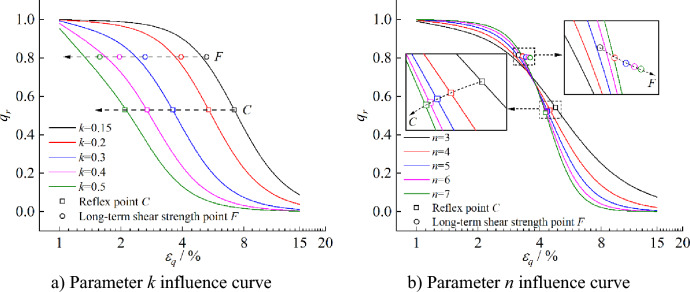
Influence curves of isochronous stress ratio-logarithmic strain curve parameters* k* and *n* on creep mechanical feature points *C* and* F.*

Based on feature points *C* and* F* on the isochronous stress ratio-logarithmic strain curve, the effects of curve model parameters* k* and* n* on the curve feature points are analyzed, as shown in Fig. 12. It can be observed that under the influence of the curve model parameter* k*, the curve feature points* C* and* F* move parallel to the strain axis as* k* increases, indicating that the parameter *k* only affects the strain at the feature point but not the stress at the feature point. Under the influence of the curve model parameter* n*, the strain at point *C* increases while the stress decreases as the parameter* n* increases—meanwhile, the strain and stress at point *F* nonlinear decreases. Therefore, the isochronous stress ratio-logarithmic strain curve is more sensitive to model parameters* n*.

## Conclusion

Based on Chengdu clay creep tests, the isochronous stress ratio-logarithmic strain curve and its model were proposed in this study, and a creep model reflecting the whole clay creep process was established. The specific conclusions are as follows.

1. The Chengdu clay creep tests revealed that the creep deformation of Chengdu clay included transient deformation, decay creep deformation, steady-state creep deformation, and accelerated creep deformation. Based on the isochronous stress–strain curve, an isochronous stress ratio-logarithmic strain curve, which had significant concave-convexity and boundary properties, was proposed. The mathematical model of the isochronous stress ratio-logarithmic strain curve is proposed from the phenomenological perspective, and the calculation methods for the various creep mechanical feature points were provided, including the linear elastic extremum point, initial yield point, long-term strength point, and plastic point.
2. Based on the isochronous stress ratio-logarithmic strain curve properties and feature points, the mechanical properties of the clay loading process were considered, and linear elastic, viscoelastic, viscoplastic, and plastic elements were established. In particular, when the loading stress of the clay was between the initial yield stress and the plastic stress, the internal structure of the soil mass sustained hardening and damage at the same time. When the loading stress was below the long-term strength, the soil structure eventually stabilized, at which time the hardening effect dominated. When the loading stress was greater than the long-term strength, the soil structure failed eventually, at which time the damage effect dominated. On this basis, viscoplastic elements considering the load hardening effect and the damage effect were established.
3. Comprehensively considering the Chengdu clay creep test results and the established element model, the linear elastic element described the transient deformation upon loading, the viscoelastic element and the viscoplastic element considering the load hardening effect describe the decay creep deformation, the viscoplastic element considering the load damage effect describes the steady-state creep deformation, and the plastic element describes the accelerated creep deformation. Based on this, the clay creep model was established, and its rationality was verified by the creep test data.

## Data Availability

Some or all data, models, or code that support the findings of this study are available from the corresponding author upon reasonable request.
